# Use of Ultrasonication Technology for the Increased Production of Plant Secondary Metabolites

**DOI:** 10.3390/molecules22071046

**Published:** 2017-06-23

**Authors:** Md. Mohidul Hasan, Tufail Bashir, Hanhong Bae

**Affiliations:** Department of Biotechnology, Yeungnam University, Gyeongsan 38541, Korea; mhasan@hstu.ac.bd (M.M.H.); tufail.bashir1@gmail.com (T.B.)

**Keywords:** plant secondary metabolites, ultrasonication, biosynthesis, stimulation, plant response

## Abstract

Plant secondary metabolites (PSMs) provide taste, color, odor, and resistance to plants, and they are also used to treat cancer and cardiovascular diseases. Synthesis of PSMs in plants is stimulated in response to different forms of external stress. Use of ultrasonication (US) to clean or decontaminate fruits and vegetables leads to physical stress that finally results in the accumulation of PSMs. US can stimulate accumulation of taxol, ginsenoside saponins, shikonin, and resveratrol, e.g., up to 319-fold increase of resveratrol synthesis has been observed in grape due to US. US also increases carotenoids, total phenolics, and isoflavonoids accumulation. Furthermore, US shows synergistic effects in PSMs synthesis-when combined with ultraviolet (UV) irradiation, jasmonic acid (JA) or salicylic acid (SA). It has been observed that US stimulates the production of reactive oxygen species (ROS) which then upregulates expression of phenylalanine ammonia lyase (*PAL*), resulting in the synthesis of PSMs. In this review, we summarize the effects of US, as a physical stress, to maximize the accumulation of PSMs in crop produce and in cell cultures.

## 1. Introduction

Plant secondary metabolites (PSMs) impart taste, color, and odor as well as provide resistance to the plants against pathogens [1]. Consumption of food enriched in PSMs has a beneficial role for human health because of their potential antiradical (scavenging) activities [2]. PSMs are classified into three major groups i.e., terpenes, alkaloids and phenolic compounds, based on their synthesis from different metabolic pathways [3]. Terpenes constitute the largest group of PSMs which are synthesized via the mevalonate (MVA) and methylerythritol 4-phosphate (MEP) pathways [3]. Some important terpenes include gibberellins, carotenoids, abscisic acid, etc., which play an important role in plant growth and development [3,4]. Alkaloids have been used as drugs to treat malaria and cancer, and they also offer crops protection against pathogens [5]. Phenolic compounds include flavonoids, non-flavonoids, and isoflavonoids, which play a significant role in plant growth, reproduction and defense [4,6]. However, some important PSMs such as taxol and resveratrol have already shown their effectiveness in treating different types of human cancers and cardiovascular diseases, and even act as anti-aging agents [7,8]. To thwart cancer, taxol induces cell death and blocks mitosis at very low concentrations (10 nM) [9]. Because of the plethora of biological functions, PSMs have been used since ancient times in traditional medicines and also in different industries such as cosmetics, fine chemicals, and more recently in nutraceuticals [10]. Currently, PSMs are available in the market as herbal or dietary constituents in the form of capsules, tablets or powder, and the demand for beneficial PSMs produced from natural sources is increasing worldwide [11]. However, there still exists a paucity of PSMs from natural synthesis.

Synthesis of PSMs in plants is affected by biotic and abiotic factors, including pathogens, light, UV radiation, wounding and nutrient deficiencies [12,13]. In response to a stimulus from different forms of external stress, many transcription factors-that are responsible for the synthesis of different PSMs-are regulated [14]. Moreover, it has been observed that the enhanced accumulation of PSMs is directly linked with up- or down-regulation of enzymes, responsible for the synthesis of specific metabolites, e.g., synthesis of resveratrol by PAL enzymes [14].

Ultrasonic cleaning baths have been used for the application of US at 20–400 kHz, during the cleaning and decontamination of fruits and vegetables [15]. Subjecting fruits and vegetables to US can induce production of beneficial PSMs, without affecting their surface qualities [16,17]. For instance, dramatic rise (1.5–319 fold) in the accumulation of different PSMs including resveratrol, taxol, saponins, and shikonin has been reported after US treatment [14,18,19,20,21,22,23]. In light of the above reports, further investigations on the induction and accumulation of PSMs in response to the application of US, or its combinatorial use with other forms of synergistic stresses, is warranted. Therefore, in this review, we summarize the past findings on PSMs accumulation by using US, or US in combination with SA and JA.

## 2. Ultrasonication Induces Biosynthesis of Terpenoids

### 2.1. Saponins

**Figure 1:**
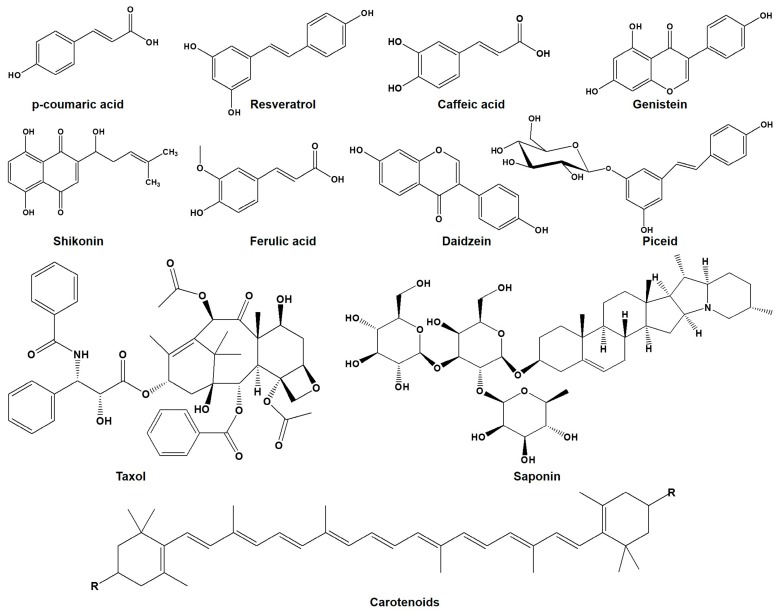
Chemical structure of different plant secondary metabolites (PSMs) induced by ultrasonication (US) or its combined use with ultraviolet ray (UV) or with methyl jasmonate (MJ)/salicylic acid (SA).

Ginseng (*Panax ginseng*) contains a large number of beneficial compounds including ginsenosides saponins (Figure 1) [24]. Treatment of cell suspension cultures of ginseng with low power US up-regulates synthesis of saponins (Table 1) [22]. As expected, depression in the growth and viability of ginseng cells was observed immediately after US treatment, possibly due to the mechanical stress of US. After this, recovery and resumption of the growth patterns of the ginseng cells were quite similar to the growth of the cells in the normal culture. Nevertheless, slightly higher biomass of ginseng cells was noticeable in the ambience of low power US, when compared with the cells of the normal culture. Interestingly, cell growth and saponins yields, show a significant correlation with US power and its exposure time [22]. Such effects could be the result of the mechanical stress or microstreamings, arising due to acoustic cavitation, following the use of US. Cavitations from low-intensity US might increase the cell membrane permeability and also denaturation of DNA and proteins [25,26]. Moreover, microstreaming can cause shear stress and increased mass transfer, which might stimulate metabolic activity in cells [25]. Hydrogen peroxide (H_2_O_2_) production by US treatment can also explain the stimulation of saponins synthesis, as an US-induced plant defense response [22].

### 2.2. Shikonin

Shikonin (Figure 1) is a hemiterpenoid produced by *Lithospermum erythrorhizon* cells through shikimate phenylalanine and isoprenoid pathways [27]. Moderate change in the enhancement of shikonin yield has been detected due to low power US treatment in a time-dependent fashion [23]. Moreover, increase in the cellular release of shikonin arises from perturbation of membrane permeability due to the US. However, US treatment in a two-phase cell culture system, also improves the shikonin yield, as seen by its more than three-fold rise (Table 1) [23]. The underlying mechanism for this effect has been attributed to the stimulation of phenylalanine ammonia lyase (*PAL*) and p-hydroxybenzoic acid geranyltransferase, after US treatment [23]. However, mass transfer effects of US, along with stimulation of key enzymes of shikonin synthesis and US-induced plant defense response, might also explain the reasons for the enhanced production of secondary metabolites in plant cells.

### 2.3. Taxol

Taxol is an important anti-cancer agent derived from the Pacific yew tree (Figure 1) [28]. *Taxus* plant grows very slowly and taxol recovery from its bark is very low [29]. However, sustained studies for the increased production of taxol have been carried out for decades with some success and even studies with cell culture system to enhance the taxol yields have borne some fruit [30]. Low power and short exposure of US treatment in cell suspension cultures of *Taxus chinensis* can potentially increase the accumulation of taxol (Table1) [31,32]. US might elicit taxol synthesis by the transient production of O_2_ and H_2_O_2_, which can induce expression of enzymes involved in jasmonic acid (JA) synthesis [32]. Suppression of reactive oxygen species (ROS) and JA accumulation by putative ROS scavengers and lipoxygenase (LOX) inhibitor, respectively, inhibits taxol production, which implies that oxidative burst and JA signals can play an important role in taxol synthesis [32]. In addition, use of methyl jasmonate (MJ) in combination with US shows synergistic effects for eliciting taxol synthesis (Table 1) [31].

Although SA addition to cells induces higher amounts of taxol in comparison to taxol synthesis after US, US enhances the effect of SA addition on the taxol yields [21,33]. In addition, higher production of H_2_O_2_ occurs by both the procedures, i.e., SA addition and US on the cells, which also results in increased accumulation of membrane lipid peroxidation and malondialdehyde (MDA), ultimately facilitating synthesis and release of taxol [21,33]. However, formation of membrane lipid peroxides due to higher ROS activity may also trigger phenylpropanoid defense responses in a cell [34]. In addition, there may be a link between US induced *PAL* expression and nitric oxide synthase (NOS)-as NOS activity leads to higher ROS (H_2_O_2_) and *PAL* activity is also positively regulated by ROS-because suppression of NOS can also inhibit *PAL* gene expression [35].

### 2.4. Carotenoids

Carotenoids are tetraterpenoids which have antioxidants properties, protect against cellular damage and chronic diseases and also impart color to plants (Figure 1) [36]. US significantly increases carotenoid accumulation in carrot and apple juice [37,38]. Interestingly, dry carrot displays higher accumulation of carotenoids than fresh carrot after US treatment [35]. However, optimum treatment of US might more effectively prevent degradation of carotenoids in dry carrots compared to fresh one [39]. Destruction of cellulose due to US might facilitate release of compounds from cells, which ultimately results in the accumulation of carotenoids [37]. Heat produced from mechanical events due to US may also aid in breaking the complex carotenoids and proteins, which might help in the enhanced extrusion of carotenoids from cells [40].

## 3. Ultrasonication Induces Biosynthesis of Polyphenols

### 3.1. Total Phenolics

**Figure 2:**
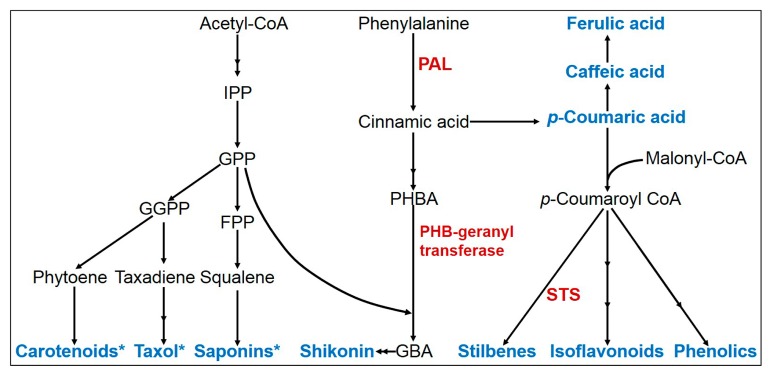
Biosynthesis pathway describes ultrasonication-mediated up-regulation of secondary metabolites (in blue) and some major enzymes (in red). *, no available report for differential regulation of enzymes synthesizing these compounds. Abbreviations: IPP, Isopentenyl Diphosphate; GPP, Geranyl Diphosphate; GGPP, Geranylgeranyl Diphosphate; FPP, Fernesyl Diphosphate; PHBA, *p*-hydroxybenzoic acid; GBA, 3-geranyl-4-hydroxybenzoic acid; *PAL*, Phenylalanine Ammonia Lyase; *STS*, Stilbene Synthase.

Herbal products often have antioxidative and pharmacological effects due to the presence of phenolic compounds, which makes them of paramount medicinal importance for daily home use [11]. Although high power US causes cell disruption and death, it also increases the accumulation of phenolic compounds. However, low energy US has shown a potential role in increasing the total phenolic content in different vegetables including carrot (*Daucus carota*), lettuce and peanut kernel (Table 1) [41,42,43,44]. Other than the crop system, low power US also enhances total phenolics in cell culture systems of yew tree or ginseng (Table 1) [21,45]. Surprisingly, no significant change in antioxidant activities or phenolic content occurs after US treatment in sliced or ground peanut kernels, probably because of the variations in treatment and subsequent incubation time [14,18]. However, induction of phenolic content in vegetables by low energy US depends on the subsequent incubation time, and this process does not have any negative effects on the aesthetic looks of the vegetables [44]. Ultrasonication stress generates ROS, which helps in stimulating the synthesis of phenolic compounds in vegetables [44]. Moreover, US treatment increases the respiration rate in carrot, which results in higher ROS levels, and this increase of ROS production up-regulates expression of the *PAL* gene (Figure 2) [43,45].

Synergistic effect of US with UV irradiation or SA on increased synthesis of total phenolics has been observed in peanut kernels, yew tree and hazelnut cell cultures [21,33,41,42]. However, this phenolic accumulation response also depends on the SA concentration and the incubation time [41,42]. The role of SA as a signaling compound for the induction of plant defense and secondary metabolite synthesis in cell culture system is well established; however, synergistic effects of US and SA towards the increased production of phenolic compounds arises due to the induction of H_2_O_2_ accumulation [46,47].

### 3.2. Stilbenes

Stilbenes include many compounds such as resveratrol and its glucoside piceid, and they are known to play an important role against cardiovascular diseases and cancer (Figure 1) [8]. As only a few plant species synthesize resveratrol-peanut and grape have relatively higher amounts-processes such as US, which can enhance its levels, gain importance. Cleaning during US can dramatically increase the resveratrol levels in peanut kernel, grape skin, grape leaves and even in grape juice (Table 1) [14,18,19,20,48,49]. Low power US with short-term exposure is more effective in eliciting accumulation of resveratrol [18,49]. Accumulation of resveratrol also depends on the duration of US treatment along with the subsequent undisturbed incubation period of the samples, for 3–12 h [14]. Nevertheless, it is expected that increased content of resveratrol due to US possibly happens by the transcriptional upregulation of resveratrol synthase (RS) (Figure 2) [14].

Synergistic effects of US and UV increase resveratrol in imbibed or roasted peanut kernels [42,48]. Moreover, use of US in the sliced peanut induces higher levels of resveratrol and piceid, when compared with UV irradiation alone (Table 1) [19]. However, the combined effect of US and UV on stilbene accumulation depends on the exposure time and distance from the UV source [19]. Interestingly, low levels of allergic proteins can also be achieved with US treatment in resveratrol-enriched peanut sprouts [50]. UV induces the expression of *PAL* and stilbene synthase (*STS*) simultaneously, which can play a role in the increased accumulation of stilbene compounds (Figure 2) [51]. Similarly, US also induces the elevation of *PAL* gene expression, and PAL protein function in deamination of phenylalanine for coumaryl CoA biosynthesis, which is the precursor of resveratrol [45,52]. However, it is not yet clear how US and UV work synergistically to stimulate synthesis of resveratrol [48].

### 3.3. Hydroxycinnamic Acids

*p*-Coumaric acid, ferulic acid, and caffeic acid are hydroxycinnamic acid derivatives of polyphenolic compounds (Figure 1) [53]. These compounds have strong antioxidant and anti-inflammatory effects, and are also used to treat diabetes and obesity-related health disorders [54]. Synthesis of *p*-Coumaric acid and ferulic acid is induced in peanut kernels by US (Figure 2). In comparison to US, synthesis of higher amounts of *p*-Coumaric and ferulic acid was seen under the UV irradiation [42]. Similar to the increased stilbene synthesis, US and UV when combined together display synergistic effects towards the increased accumulation of coumaric acid, ferulic acid and caffeic acid (Table 1) [42]. It is also pertinent to say that US alone or in combination with UV has a great impact on the accumulation of different types of hydroxycinnamic acid in peanut kernels, and this might help in replacing red wine with roasted peanuts as a source of those phenolic compounds.

### 3.4. Isoflavonoids

Because of the presence of isoflavonoids (daidzein or genistein), *Genista tinctoria* plays an important role in combating several diseases including hypoglycemia, inflammatory disorders, and different types of cancer (Figure 1) [55]. Cell suspension culture of *G. tinctoria* treated with US, exhibits increased daidzein and genistin contents (Table 1 and Figure 2) [56]. Moreover, the time of treatment and subsequent incubation time also influence the accumulation of both compounds. Fluid motion and microstreaming arising from the acoustic cavitation effects of US might cause such stress related bio-effects on the cells growing in liquid media and this might also increase the production of secondary metabolites and induce defense responses [57].

## 4. Conclusions and Future Prospects

Low power US offers an inexpensive and simple tactic for increasing the accumulation of beneficial PSMs along with cleaning and decontamination of the crop produce. Moreover, the strategy is also effective and feasible for stimulation of beneficial compounds in cell cultures. The combined use of US with UV or JA/SA enhances accumulation of some PSMs, in comparison to their separate use. However, accumulation of PSMs also depends on the incubation period after US or combined treatment procedures, as it might allow sufficient time to reach a threshold state for the activation of the required genes, which trigger such effects. Therefore, it is important to understand underlying mechanistic details by identification of key molecular players involved in induction and accumulation of PSMs due to combined effects of US, UV or JA/SA.

Researchers have tried to optimize US methods in the field of food processing or extraction of metabolites. However, continuous in-depth studies are required to integrate a completely automated ultrasound system, which is low cost and energy efficient, to achieve large-scale production of beneficial secondary metabolites in the crop produce. Plants contain diverse groups of beneficial compounds; therefore, it is essential to study the effects of US on such compounds. For instance, it would be interesting to study if US affects alkaloid production because, to our knowledge, there are no such reports in the literature. In addition, the use of US procedures alone or in combination with other available and feasible physical stressors or elicitors should be investigated to explore the maximum production of PSMs.

## Figures and Tables

**Table 1:** Accumulation of plant secondary metabolites (PSMs) in response to the use of ultrasonication (US) or its combined effect with ultraviolet radiations (UV) or methyl jasmonate (MJ)/salicylic acid (SA).

PSMs	Cell Culture/Produces	Treatment (US, US + MJ/SA)	Treatment Time (min)	Induced by	References
Saponins	*Panax ginseng*	35.5 KHz (82 mW/cm^3^)	1–4	∼1.8-fold	[22]
Shikonin	*Lithospermum erythrorhizon*	38.5 KHz (113.9 mW/cm^3^)	1–8	∼1.7-fold	[23]
Taxol	*Taxus* tree	38.5–40 KHz (3.5–55.6 mW/cm^3^)	2	2-fold	[21,31]
		US + MJ 60 mM	2	2.5-fold	[31]
		US + SA 50 mg/L	2	8-fold	[21]
	*Corylus avellana*	40 kHz (4.91 W/cm^2^)	2–10	2-fold	[33]
		US + SA 50 mg/L	3	14-fold	
Carotenoids	*Daucus carota*	35 KHz (4W/cm^2^)	10–30	1.5-fold	[37]
	*Malus* juice	25 KHz (2W/cm^2^)	60	∼1.4-fold	[38]
Resveratrol	*Vitis* fruit skin	40 kHz	5	8-fold	[14]
	*Vitis* leaves	40 kHz	15	1.9-fold	
	*Vitis* fruit juice	40 kHz	5	1.5-fold	[20]
	*Arachis hypogaea* kernel	20–25 KHz (25–120 mW/cm^3^)	2–8	∼319-fold	[18,19,48]
		US + 254 nm UV	(2–8) + (10–30)	∼211-fold	[19,41,48]
	*Arachis hypogaea* sprout	100 KHz	20	∼3.34-fold	[50]
Piceid	*Arachis hypogaea* kernel	20 KHz (25–75 mW/cm^3^)	2–8	∼213-fold	[19]
		US + UV	(12 + 10)	2.5-fold	[42]
Total stilbenes	*Arachis hypogaea* kernel	20 KHz (25–75 mW/cm^3^)	2–8	∼97-fold	[19]
Total phenolics	*Arachis hypogaea* kernel	39.2 mW/cm^3^	4	2.2-fold	[42]
		US + UV	(4 + 10)	1.5-fold	
	*Panax ginseng*	38.5 kHz (14 to 61 mW/cm^3^)	2	5-fold	[45]
	*Daucus carota*	24 kHz	5	∼2.3-fold	[43]
	*Lactuca sativa*	25 kHz	1–3	∼1.4-fold	[44]
	*Taxus* tree	40 KHz	2	2-fold	[21]
		US + SA 50 mg/L	2	2.5-fod	
	*Corylus avellana*	40 kHz (4.91 W/cm^2^)	3	1.2-fold	[33]
*p*-Coumaric acid	*Arachis hypogaea* kernel	40 mW/cm^3^	8	4-fold	[41]
		US + UV	(8 + 10)	7-fold	
Ferulic acid		40 mW/cm^3^	12	14-fold	
		US + UV	(8 + 10)	24-fold	
Caffeic acid		US + UV	(8 + 10)	4.2-fold	
Daidzein	*Genista tinctoria*	35 KHz (0.1 mW/cm^3^)	5	2-fold	[56]
